# Propensity score matching analysis to comparing cisplatin versus nedaplatin based doublet agent concurrent chemoradiotherapy for locally advanced cervical cancer

**DOI:** 10.1038/s41598-023-36433-5

**Published:** 2023-06-08

**Authors:** Yue Zhang, Shasha Fan, Minjie Shan, Wen Zou, Yeqian Feng, Tao Hou, Xianling Liu, Jingjing Wang

**Affiliations:** 1 Department of Oncology, The Second Xiangya Hospital of Central South University, 139 Middle Renmin Road, Changsha, 410011 Hunan People’s Republic of China; 2 Department of Oncology, The First Affiliated Hospital of Hunan Normal University, Hunan Provincial People’s Hospital, Hunan, People’s Republic of China

**Keywords:** Chemotherapy, Radiotherapy, Cervical cancer

## Abstract

This study evaluated the efficacy and safety of cisplatin and nedaplatin in three-week doublet agent concurrent chemoradiotherapy (CCRT) for patients with locally advanced cervical cancer (LACC). We retrospectively enrolled patients with stage IIB-IIIC2 cervical cancer who received doublet agent CCRT from January 2015 to December 2020. Clinical outcomes were analyzed using the Kaplan–Meier method and a Cox proportional hazards model. Propensity score (PS) matching analysis was used to compare cisplatin plus docetaxel group and nedaplatin plus docetaxel group. A total of 295 patients were included. The 5-year overall survival rate (OS) and progression free survival rate (PFS) were 82.5% and 80.4%, respectively. After PS matching, there were 83 patients each in the nedaplatin group and cisplatin group. There were no significant differences in objective response rates (97.6% and 98.8%, p = 0.212), 5-year OS rate (96.5 vs 69.8, p = 0.066), PFS rate (90.8 vs 72.4, p = 0.166), and toxicity between the two groups. Doublet agent concurrent chemoradiotherapy is feasible, safe, and shows high efficacy in LACC patients. Here, cisplatin group has a trend of better prognosis, suggesting that cisplatin is preferred and nedaplatin can be considered for replacement when cisplatin is intolerant.

## Introduction

Cervical cancer, as the most common gynecologic malignancy, is the fourth female malignant tumor in the world in terms of female morbidity and mortality^1^, and its treatment remains a challenge. About 45% patients are diagnosed with locally advanced cervical cancer (LACC), including FIGO stage IIB-IVA, and the standard treatment was platinum-based concurrent radiotherapy and chemotherapy (CCRT)^2,3^. However, the use of platinum-based dual therapy or platinum single-dose therapy in CCRT is still controversial^4,5^. Multiple meta-analyses showed that compared with radiotherapy plus platinum single-dose therapy, radiotherapy plus platinum dual therapy could significantly prolong the prognosis of patients with LACC, but the incidence of adverse events would also increase^6–8^. At present, there are many platinum drug options, such as cisplatin, carboplatin, nedaplatin, etc. Nedaplatin is a second-generation platinum analogue, as a derivative of cisplatin, which was approved for the first time in Japan in 1983. Its solubility in water is about ten times that of cisplatin, and its digestive and renal toxicity is lower than that of cisplatin^9^10]. A meta-analysis comparing single-agent CCRT showed that nedaplatin group had a better ORR and lower chemotherapy toxicity compared with cisplatin group^11^. Another phase III randomized, controlled trial showed the 3-year OS rate of nedaplatin group was similar with cisplatin group, and the gastrointestinal reaction was milder, and the hepatotoxicity was heavier compared with cisplatin group^12^. However, a study of propensity score matching has different results: the recurrence rate of nedaplatin group was significantly higher, and the 3-year PFS rate was lower compared with cisplatin group. The 3-year OS rate and grade 3 to 4 toxicity were similar between the nedaplatin and cisplatin groups^13^. The above studies are to explore the efficacy and safety of platinum single drug weekly therapy, and the results are controversial. In the field of platinum based dual reagent CCRT, there are fewer studies comparing the efficacy of different platinum drugs. This study aims to evaluate the efficacy and safety of cisplatin and nedaplatin in doublet agent CCRT.

## Materials and methods

### Patients

A cohort of patients diagnosed with cervical cancer were retrospectively examined at the Second Xiangya Hospital of Central South University and the Hunan Provincial People’s Hospital from January 2015 to December 2020. The following patients were included in the study: (1) those with a pathological diagnosis of cervical squamous cell carcinoma, adenocarcinoma, or adenosquamous carcinoma, (2) those with stage IIB-IIIC2 cervical cancer (according to the FIGO 2018 staging system) who received platinum-based doublet agent CCRT, (3) those whose follow-up data were complete, and (4) those with IMRT. The following patients were excluded from the study: (1) those with other tumors, infectious diseases, hematological diseases, any mental disorder or somatic comorbidities, severe liver or renal dysfunction, or uncontrolled life-threatening illness, (2) those who received previous radiotherapy and/or chemotherapy; (3) those who had distant metastasis; or (4) those who were pregnancy or lactation. The last follow-up was conducted on August 31, 2022. All methods were performed in accordance with the relevant guidelines and regulations, and informed consent was obtained from all subjects and/or their legal guardian(s). The study was conducted in accordance with the Declaration of Hensinki and approved by the appropriate ethics review board of the Second Xiangya Hospital of Central South University, Changsha, China.

### Treatment

All patients underwent CCRT, and some patients with good tolerance also received adjuvant chemotherapy after CCRT. Chemotherapy protocols were selected according to the patients' renal function and physical status. Patients received concurrent chemotherapy with docetaxel (75 mg/m^2^) combined with carboplatin (AUC = 4–5)/cisplatin (50–70 mg/m^2^)/nedaplatin (50–75 mg/m^2^), once every three weeks. So, patients were divided into cisplatin group, carboplatin group and nedaplatin group. The patient's whole blood cell count, renal function, and liver function were routinely monitored during treatment.

Intensity-modulated radiation therapy was used for external irradiation, which was planned with the Varian Eclipse Treatment Planning System version 11.0 (Varian Medical Systems, Palo Alto, CA, USA) and delivered with 6-MV X-rays using Varian 23EX (Varian Medical Systems).

Gross tumor volume (GTV) includes positive lymph nodes, which was confirmed using computed tomography (CT), magnetic resonance imaging (MRI), or positron-emission tomography (PET), and the clinical target volume (CTV) included the regional-nodal basin. The planning target volume was delineated by margins of 5–10 mm around the GTV and CTV. Cone-beam CT was performed weekly. The CTV dose was 45–50 Gy with 1.8 or 2 Gy administered daily, and the GTV dose was 60 Gy with 2.4 Gy administered daily. Patients received 2D intracavitary brachytherapy (iridium 192) with a dose of 30 Gy/5fx to point A (defined as 2 cm lateral to the central canal of the uterus and 2 cm above the cervical opening) once a week, without EBRT treatment on the day of intracavitary treatment. The entire RT course (including EBRT and brachytherapy) was completed within 8 weeks.

### Data collection and follow-up

Clinical information, including that of age, initial stage, pathological pattern, tumor diameter, chemotherapy protocol, and imaging studies (CT, MRI, or positron emission tomography/CT) was collected. The imaging examination was carried out 2 months after the end of radiotherapy, and the treatment response was evaluated using the Response Evaluation Criteria in Solid Tumors (RECIST 1.1). Follow-up after the completion of CCRT includes every 3 months in the first 2 years, every 6 months in the next 3 years, and every year thereafter. At each follow-up visit, physical examination, imaging examination, hematology examination or cervical cytology examination are selected according to the patient's condition.

### Statistical analysis

The SPSS software (version 26.0; SPSS, Inc., Chicago, IL, USA) was used for all statistical analyses. Overall survival (OS) calculated from the date of diagnosis until the date of death or final follow‐up. Progression‐free survival (PFS) was defined as survival from the date of diagnosis until the date of (1) disease progression, (2) relapse, (3) mortality from any cause, or (4) final follow‐up.

Fisher’s exact test and χ^2^ test was used to determine whether there was a correlation between two variables. Univariate analysis was performed using the Kaplan–Meier method to assess the 5-year OS and PFS rates. Statistical differences between survival curves were evaluated using the log-rank test. Multivariate Cox proportional hazards models were created using the input selection technique of factors significant in the univariate analysis. Patients were classified into the cisplatin group and nedaplatin group. Treatment outcomes were compared between the two groups after 1:1 ratio propensity score (PS) matching with the nearest neighbor matching method using calipers of width equal to 0.02 of the SD of the logit of the PS, and adjusted for age, stage, BMI, pathological pattern, tumor diameter, differentiation, complication, and chemotherapy cycles^14^. In all tests, P-values < 0.05 indicated a significant difference. Hazard ratios (HRs) and their 95% confidence intervals (CIs) were estimated to assess the magnitude of risk. And I confirm that all methods were performed in accordance with the relevant guidelines and regulations.

### Institutional review board statement

The study was conducted in accordance with the Declaration of Hensinki and approved by the appropriate ethics review board of the Second Xiangya Hospital of Central South University, Changsha, China.

## Results

### Patient characteristics

A total of 295 patients enrolled. The median follow-up time was 53 months (20–92 months). The median age was 55 years (32–73 years). The median number of chemotherapy cycle is 4 (1–6 cycles). There were 44 patients received concurrent chemotherapy with median cycle of 2, and 251 patients received concurrent and adjuvant chemotherapy with median cycle of 4. There were 198 patients were treated with nedaplatin, 87 with cisplatin and 10 with carboplatin. In the nedaplatin group, 11 patients only received one cycle of chemotherapy because they could not tolerate chemotherapy. In the cisplatin group, 3 patients only received one cycle of chemotherapy. Patients stopped chemotherapy if they couldn't tolerate it, and no patients received reduced dose chemotherapy. Patients’ characteristics are summarized in Table 1. All patients completed external irradiation, while only 8 patients did not complete brachytherapy. Among them, 5 patients were unable to tolerate adverse reactions, and 3 patients were due to family and social reasons.

**Table 1:** Clinical characteristics of patients.

Variable	Number (%)	Variable	Number (%)
**Total**	295 (100)	Differentiation	
Median age (years)	55	High	62 (21.0)
Median BMI	22.66	Medium	127 (43.1)
Figo stage		Low	106 (35.9)
IIB	118 (40.0)	Complication	
IIIA	14 (4.7)	Hypertension	50 (16.9)
IIIB	85 (28.8)	Diabetes	23 (7.8)
IIIC1	59 (20.0)	Others	38 (12.9)
IIIC2	19 (6.4)	Chemotherapy cycles	
Pathology		1–3	44 (14.9)
Squamous carcinoma	280 (94.9)	4–6	251 (85.1)
Adenocarcinoma	13 (4.4)	Chemotherapy	
Adenosquamous carcinoma	2 (0.7)	Carboplatin	10 (3.4)
Tumor diameter		Cisplatin	87 (29.5)
< 4 cm	131 (44.4)	Nedaplatin	198 (67.1)
≥ 4 cm	164 (55.6)	Radiotherapy	
		Conventional radiotherapy	217 (73.56)
		Extended-field radiotherapy	78 (26.44)

### Prognostic analysis and propensity score analysis

**Figure 1:**
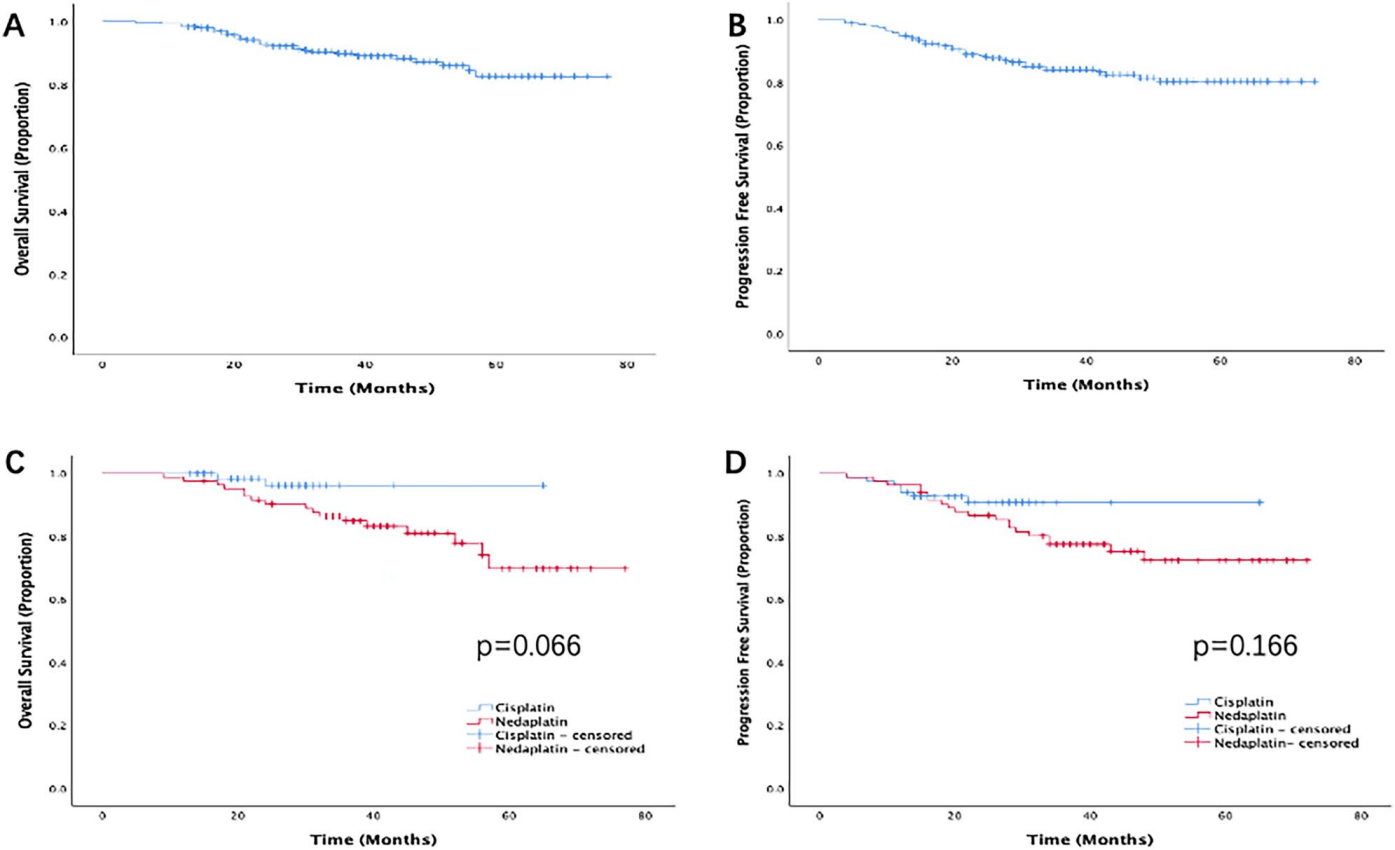
(**A**) Overall survival and (**B**) progression-free survival of patients with LACC who received doublet agent concurrent chemoradiotherapy. (**C**) Overall survival and (**D**) progression-free survival of cisplatin group and nedaplatin group after PS matching.

All patients received doublet agent concurrent chemoradiotherapy, and the 5-year OS and PFS rates were 82.5% and 80.4%, respectively (Fig. 1A,B). In the univariate analysis, chemotherapy protocol, and therapy response significantly influenced the OS, and only therapy response significantly influenced the PFS rates (Table 2). The stage was divided into stage II, stage IIIA/B, and stage IIIC for stratified analysis. It was found that there was a significantly difference in OS between stage II and stage IIIc (OS: 87.9% vs. 70.1%, p = 0.045) (Supplemental Table S1). Patients with tumor diameter < 4 cm had better PFS trend than those who ≥ 4 cm (PFS: 86.2% vs. 75%, p = 0.054). The above variables with statistical significance in univariate analysis, such as stage, tumor diameter, and chemotherapy, were included in multivariate analysis, and no independent prognostic factors were found (Table 3).

**Table 2:** Univariate analysis of overall survival, progression-free survival rates and objective response rates for all patients.

Variable	Univariate analysis			
	5-year OS rate (%)	P-value	5-year PFS rate (%)	P-value
Total	82.5		80.4	
Age, years				
≤ 55	82.5		76.4	
> 55	81.8	0.192	85.5	0.199
BMI				
< 22.66	82.2		79.0	
≥ 22.6	82.7	0.315	80.7	0.102
Figo stage				
IIB	87.9		84.6	
IIIA/B	81.7		79.6	
IIIC	70.1	0.086*	75.5	0.193
Pathology				
Squamous carcinoma	82.4		79.8	
Adenocarcinoma	79.1		88.9	
Adenosquamous carcinoma	100	0.725	100	0.664
Tumor diameter				
< 4 cm	85.7		86.2	
≥ 4 cm	79.8	0.119	75.0	0.054
Differentiation				
High	74.6		71.7	
Medium	83.2		86.4	
Low	84.3	0.238	79.2	0.167
Complication				
Yes	80.2		74.4	
No	84.1	0.483	84.1	0.235
Chemotherapy cycles				
1–3	82.9		77.4	
4–6	82.2	0.338	81	0.743
Chemotherapy				
Carboplatin	64.0		70.0	
Cisplatin	95.2		89.9	
Nedaplatin	82.1	0.017	80.2	0.208
Efficacy evaluation				
CR	97.4		96.8	
PR	69.4		69.1	
SD	45.7	0.000	15.0	0.001

**Table 3:** Responses and survival outcomes before and after matching.

Variables	Before matching			After matching		
	Cisplatin group (n = 86)	Nedaplatin group (n = 198)	P	Cisplatin group (n = 83)	Nedaplatin group (n = 83)	P
CR	50	93		50	40	
PR	34	98		31	42	
SD	2	7	0.218	2	1	0.212
ORR (%)	97.7	96.5		97.6	98.8	
OS						
5-y OS (%)	95.2	82.0	0.291	96.5	69.8	0.066
PFS						
5-y PFS (%)	89.8	80.1	0.645	90.8	72.4	0.166

Patients who received carboplatin had worser OS than those who received cisplatin and nedaplatin (OS: 64% vs. 95.2% vs. 82.1%, p = 0.017). Then comparing the prognosis difference between cisplatin and nedaplatin group. The clinical characteristics of the cisplatin group and nedaplatin group were compared. It was found that pathology and tumor diameter between the two groups were not well-balanced (Supplementary Table S2). After PS matching, there were 83 patients each in the cisplatin group and nedaplatin group. All background factors between the two groups were well-balanced (Supplementary Table S2). Responses and survival outcomes before and after matching are summarized in Table 4. There was no significant difference between cisplatin group and nedaplatin group after matching in terms of ORR (97.6% vs. 98.8%, p = 0.212), 5-year OS rate (96.5% vs. 69.8%, p = 0.066), and PFS rate (90.8% vs. 72.4%, p = 0.166), however in the survival curve, it can be found that cisplatin group has a better prognosis trend than nedaplatin group (Fig. 1C,D).

**Table 4:** Multivariate analysis of clinical outcomes.

Variables	Multivariate analysis		
	HR	95% CI	P value
OS			
Figo stage	1.497	0.898–2.255	0.069
Tumor diameter	1.423	0.662–3.136	0.133
Chemotherapy	1.117	0.1555–2.248	0.757
PFS			
Figo stage	1.263	0.864–1.845	0.228
Tumor diameter	1.592	0.829–3.054	0.162
Chemotherapy	1.067	0.604–1.886	0.823

### Toxicity

In total, 85.4% of patients experienced hematologic toxicity, of which 46.1% had grade 3–4 hematologic toxicity; 59.3% had nausea/vomiting, of which 10.5% had grade 3–4 nausea/vomiting; 47.8% had radiation proctitis, 28.5% had radiation cystitis, and no patients had grade 3–4 radiation proctitis or cystitis.

After PS matching, there was no significant difference between cisplatin group and nedaplatin group in terms of hematotoxicity (85.5% vs.79.5%, p = 0.307), grade 3–4 hematotoxicity (36.0% vs.46.9%, p = 0.209), grade 3–4 neutropenia (34.9% vs.44.6%, p = 0.268), thrombocytopenia (53% vs.51.8%, p = 0.488), grade 3–4 thrombocytopenia (21.7% vs.16.9%, p = 0.431), grade 3–4 anemia (7.2% vs. 1.2%, p = 0.053), nausea and vomiting (63.9% vs.54.2%, p = 0.207), grade 3–4 nausea and vomiting (13.3% vs.6.0%, p = 0.115), radiation proctitis (48.2% vs.45.9%, p = 0.756), and radiation cystitis (33.7% vs.22.9%, p = 0.121). However, in terms of neutropenia (78.3% vs.71.1%, p = 0.003) and anemia (83.1% vs.79.5%, p = 0.003), the cisplatin group was greater than the nedaplatin group (Table 5).

**Table 5:** Adverse event profiles.

Adverse effect	Patients n (%)	Before matching			After matching		
		Cisplatin group (n = 86), n (%)	Nedaplatin group (n = 198), n (%)	P	Cisplatin group (n = 83), n (%)	Nedaplatin group (n = 83), n (%)	P
Hematologic	253 (85.4)	74 (86.0)	171 (86.4)	0.943	71 (85.5)	66 (79.5)	0.307
G3-4	136 (46.1)	31 (36.0)	101 (51.1)	**0.020**	31 (36.0)	39 (46.9)	0.209
Neutropenia	219 (74.2)	59 (65.1)	148 (74.7)	**0.000**	65 (78.3)	59 (71.1)	**0.003**
G3-4	125 (42.4)	30 (34.9)	92 (46.5)	0.070	30 (34.9)	37 (44.6)	0.268
Thrombocytopenia	169 (57.3)	47 (54.7)	117 (59.1)	0.363	44 (53.0)	43 (51.8)	0.488
G3-4	52 (17.6)	18 (20.9)	33 (16.7)	0.390	18 (21.7)	14 (16.9)	0.431
Anemia	248 (84.1)	72 (83.7)	170 (85.9)	**0.000**	69 (83.1)	66 (79.5)	**0.003**
G3-4	10 (3.4)	6 (6.9)	4 (2.0)	**0.037**	6 (7.2)	1 (1.2)	0.053
Nonhematologic							
Nausea/vomiting	175 (59.3)	56 (65.1)	119 (60.1)	0.425	53 (63.9)	45 (54.2)	0.207
G3-4	31 (10.5)	11 (12.8)	20 (10.1)	0.504	11 (13.3)	5 (6.0)	0.115
Radiation proctitis	141 (47.8)	42 (48.8)	98 (49.5)	0.919	40 (48.2)	38 (45.9)	0.756
G3-4	0	0			0	0	
Radiation cystitis	84 (28.5)	28 (32.6)	56 (29.8)	0.468	28 (33.7)	19 (22.9)	0.121
G3-4	0	0			0	0	

Significant values are in bold.

## Discussion

Platinum-based CCRT is the standard treatment for patients with locally advanced cervical cancer. The main function of platinum-based chemotherapy is to increase the sensitivity of radiotherapy and eradicate micrometastasis^15^. At present, the most recommended regimen of concurrent chemotherapy is weekly cisplatin monotherapy^16^. However, the effect of cisplatin monotherapy is limited, and the 5-year OS rate in patients with IIB-IVA stage was approximately 60%^2^. Therefore, many studies have focused on platinum-based dual-agent CCRT with synergistic effects. A meta-analysis compared the efficacy and safety of RT with cisplatin monotherapy and RT with platinum-based doublet therapy in patients with locally advanced cervical cancer. The results showed that RT with platinum-based doublet therapy significantly prolonged the OS (HR 0.75, 95% CI 0.60–0.94, p = 0.01) and the PFS (HR 0.78, 95% CI 0.65–0.94, p = 0.01), but increased adverse reactions compared to the cisplatin monotherapy group^6^. Many other studies confirmed that platinum-based dual-agent CCRT has improved survival^8^. Our study evaluated the efficacy and safety of platinum-based dual chemotherapy, and the results showed that dual-agent CCRT had excellent long-term therapeutic effect, and the 5-year OS and PFS rates were 82.5% and 80.4%, respectively, which is better than cisplatin monotherapy and consistent with the current studies.

At present, many platinum-based dual-agent regimens of CCRT have been studied, A network meta-analysis enrolled 11 cohort studies on CCRT for LACC, including cisplatin monotherapy, paclitaxel monotherapy, and platinum combined chemotherapy. The results showed that cisplatin plus docetaxel might be the best choice of CCRT regimens in the treatment of LACC^17^. In addition, paclitaxel plus cisplatin^18^, gemcitabine plus cisplatin^4^, and 5-FU plus cisplatin^19^ in CCRT all showed good curative effect.

In this study, we analyzed the efficacy of carboplatin, cisplatin and nedaplatin based dual-agent concurrent chemoradiotherapy. The results showed that the prognosis of carboplatin-based CCRT is worse than that of cisplatin and nedaplatin-based CCRT. Due to the small number of patients receiving carboplatin treatment in this study, the result is controversial. A study showed that there were no statistically significant differences in recurrence and survival rates between the carboplatin CCRT group and the historical cisplatin CCRT group in 51 LACC patients with morbidity risks^20^. Another retrospective study also showed that carboplatin group have similar 3-years overall survival, progression free survival, overall response rate, and toxic effects when compared to cisplatin group^21^. However, a meta-analysis showed that carboplatin CCRT group showed a poorer tumor response and a trend toward inferior survival compared with cisplatin CCRT group. Although this is consistent with the results of this study, larger controlled studies are still needed to validate them.

Our study also showed that there is no significant difference between the cisplatin group and the nedaplatin group in terms of ORR, 5-year OS rate and PFS rate when all background factors were balanced among groups, but the cisplatin group has a better prognosis trend. Nedaplatin and cisplatin act in a similar way, both of which produce anti-tumor activity by inhibiting the replication of tumor cell DNA. However, there are still differences between these two drugs. For example, cisplatin is the substrates of membrane transporters such as hOCT1, hOCT2, and hMATE1. Nedaplatin are not transported by the transporters mentioned above. It indicates that the resistance modes of the two drugs are different, and they are not completely cross resistant. In terms of clinical application, cisplatin has a wider anti-tumor spectrum and more comprehensive data, making it the preferred drug for cervical cancer at present. An early phase 2 clinical trial showed that the CCRT regimen of paclitaxel and nedaplatin was well tolerated and effective for LACC. The 2-year PFS and OS were 82% and 93%, respectively, and 3 cases (9%) had grade 3 late complications, which suggested that concurrent chemoradiotherapy based on nedaplatin is effective and safe^22^. Another randomized phase III trial to evaluate the efficacy and safety of nedaplatin and cisplatin three-week monotherapy in CCRT showed that the 1-year PFS and OS in the nedaplatin and cisplatin groups were similar^23^. However, a meta-analysis showed nedaplatin group had higher ORR and lower toxicity than cisplatin group, which is better than other concurrent single-agent chemotherapy, such as docetaxel, paclitaxel, fluoropyrimidine, paclitaxel liposome, and irinotecan^11^. Therefore, when cisplatin is intolerant, it is feasible to use nedaplatin instead.

In terms of safety, a study showed that nedaplatin less frequently causes renal toxicity in comparison to cisplatin due to lower kidney accumulation^24^. However, there is still controversy regarding other toxicity. Our study shows that the hematological toxicity of cisplatin is slightly higher than that of nedaplatin, and there is no difference between nausea/vomiting, radiation cystitis and proctitis. However, a study showed that there were no significant differences in hematological toxicity between the nedaplatin and cisplatin for CCRT of cervical cancer, and vomiting, nausea and anorexia were more common in the cisplatin group whereas effects on liver function were more common in the nedaplatin group^12^. Another study comparing nedaplatin and cisplatin monotherapy CCRT showed that a higher frequency of grade 3–4 neutropenia, grade 1–2 anemia, thrombocytopenia in the nedaplatin group than the cisplatin group, Whereas nausea in the cisplatin group was higher than in the nedaplatin group^23^. The comparison of adverse events of nedaplatin and cisplatin in different studies has different results, which may be closely related to the enrolled population, specific treatment plans, etc., and needs to be verified by large prospective randomized clinical studies.

In terms of prognostic factor of CCRT, stage and tumor size were recognized to be closely related to the prognosis. A phase III randomized clinical trial of cisplatin monotherapy versus gemcitabine combined with cisplatin for CCRT of LACC showed that stage and large tumor size were associated with poor prognosis, regardless of treatment assigned^25^. Another study reestimated the published prognostic model of LACC and found that the reestimated prognostic factors (including FIGO stage and tumor size) in the validation sample were related to the reduction of OS and CSS^26^. Our study also showed that the stage and tumor size still affect the prognosis of patients with LACC in dual-agent CCRT. However, they are not independent prognostic factors. Whether the better effectiveness of dual-agent chemotherapy weakens their impact on prognosis needs further discussion.

Adjuvant chemotherapy after CCRT has always been controversial. A meta-analysis in 2008 and a study in 2011 suggested patients could benefit from adjuvant chemotherapy after CCRT^5,27^. In recent years, both of ACTLACC trial and OUTBACK trial evaluated the effect of adjuvant chemotherapy after CCRT, and the results showed that adjuvant chemotherapy after CCRT did not improve survival compared to CCRT alone, and the rate of grade 3–5 adverse events within one year was higher^28,29^. However, ACTLACC trial excluded patients with enlarged paraaortic lymph nodes, and OUTBACK trial did not conduct stratification analysis of clinical characteristics. Therefore, the results have limitations. A review analyzed the relevant studies of adjuvant chemotherapy and suggested that there is an absolute need for further research in order to optimally define the position of adjuvant chemotherapy in the treatment of LACC^30^. Our study showed 85.1% of patients received 4–6 cycles of chemotherapy, and the prognosis of patients receiving 1–3 cycles of chemotherapy is the same as that of patients receiving 4–6 cycles, which means that adjuvant chemotherapy did not increase the efficacy, which consistent with current mainstream research results.

There are many advances in the treatment of LACC. Immunotherapy has changed the first-line treatment mode of cervical cancer, and some studies have also been carried out in the CCRT of LACC, such as: adjuvant durvalumab with chemoradiotherapy^31^, toripalimab combined with platinum-based CCRT^32^, and pembrolizumab with chemoradiotherapy^33^ et al. The CCRT regimen of these studies is weekly cisplatin monotherapy, which are still being recruited. The study of dual-agent CCRT combined with immunotherapy has not been carried out.

This study is a double-center retrospective study. The baseline situation is complex. Although propensity score matching is used for adjustment, the population is small, which has a certain impact on the results. Especially in the prognosis analysis, the influence trend of cisplatin on prognosis was seen, but there was no statistical difference. It is necessary to further expand the sample size. In addition, there is no single-agent group for control. This study has certain limitations.

## Conclusion

Our study suggests that doublet agent concurrent chemoradiotherapy is feasible, safe, and shows high efficacy in LACC patients. Here, the cisplatin group and nedaplatin group have similar prognosis and side effects. However, cisplatin group has the trend of better prognosis, suggesting that cisplatin is preferred and nedaplatin can be considered for replacement when cisplatin is intolerant.

## Supplementary Information

Supplementary Table 1.

Supplementary Table 2.

## Data Availability

The authors agree to share anonymized data upon reasonable request by researchers. Someone wants to request the data from this study, please contact the corresponding author.
