# Supplementary material for: Propensity score matching analysis to comparing cisplatin versus nedaplatin based doublet agent concurrent chemoradiotherapy for locally advanced cervical cancer
Source: Sci Rep. 2023 Jun 8;13:9352. doi: 10.1038/s41598-023-36433-5 (PMC10250460; doi:10.1038/s41598-023-36433-5)
Supplement: Supplementary file 1 — Supplementary Table 1. [file 41598_2023_36433_MOESM1_ESM.docx]

Supplemental table 1: Stratification analysis of the effect of stage on overall survival

|  | Figo Stage | II Stage | | IIIA/B Stage | | IIIC Stage | |
| --- | --- | --- | --- | --- | --- | --- | --- |
|  |  | chi-square | P Value | chi-square | P Value | chi-square | P Value |
| Log Rank (Mantel-Cox) | II Stage |  |  | 0.139 | 0.709 | 4.028 | 0.045 |
|  | IIIA/B Stage | 0.139 | 0.709 |  |  | 2.743 | 0.098 |
|  | IIIC Stage | 4.028 | 0.045 | 2.743 | 0.098 |  |  |
