# Supplementary material for: Propensity score matching analysis to comparing cisplatin versus nedaplatin based doublet agent concurrent chemoradiotherapy for locally advanced cervical cancer
Source: Sci Rep. 2023 Jun 8;13:9352. doi: 10.1038/s41598-023-36433-5 (PMC10250460; doi:10.1038/s41598-023-36433-5)
Supplement: Supplementary file 2 — Supplementary Table 2. [file 41598_2023_36433_MOESM2_ESM.docx]

Supplementary table 2. Pretreatment characteristics before and after matching

| Characteristics | Before Matching | | | After Matching | | |
| --- | --- | --- | --- | --- | --- | --- |
|  | Cisplatin Group  (n=87) | Nedaplatin Group  (n=198) | P | Cisplatin Group  (n=83) | Nedaplatin Group  (n=83) | P |
| **Age** |  |  |  |  |  |  |
| <55 | 42 | 112 |  | 42 | 42 |  |
| ≥55 | 45 | 85 | 0.198 | 41 | 41 | 1.0 |
| **BMI** |  |  |  |  |  |  |
| < 22.66 | 45 | 95 |  | 42 | 41 |  |
| ≥22.66 | 42 | 102 | 0.608 | 43 | 40 | 1.0 |
| **Figo stage** |  |  |  |  |  |  |
| IIB | 34 | 82 |  | 32 | 22 |  |
| IIIA/B | 26 | 68 |  | 25 | 31 |  |
| IIIC | 27 | 47 | 0.432 | 26 | 30 | 0.249 |
| **Pathology** |  |  |  |  |  |  |
| Squamous carcinoma | 79 | 190 |  | 76 | 81 |  |
| Adenocarcinoma | 8 | 5 |  | 7 | 1 |  |
| Adenosquamous carcinoma | 0 | 2 | 0.031 | 0 | 1 | 0.059 |
| **Tumor diameter** |  |  |  |  |  |  |
| <4cm | 30 | 97 |  | 29 | 25 |  |
| ≥4cm | 57 | 100 | 0.027 | 54 | 58 | 0.619 |
| **Differentiation** |  |  |  |  |  |  |
| High | 24 | 36 |  | 22 | 19 |  |
| Medium | 33 | 85 |  | 33 | 35 |  |
| Low | 30 | 76 | 0.208 | 28 | 29 | 0.862 |
| **Complication** |  |  |  |  |  |  |
| yes | 35 | 70 |  | 32 | 34 |  |
| no | 52 | 127 | 0.505 | 51 | 49 | 0.751 |
| **Chemotherapy cycles** |  |  |  |  |  |  |
| 1-3 | 8 | 33 |  | 7 | 9 |  |
| 4-6 | 79 | 164 | 0.103 | 76 | 74 | 0.793 |
